# Editorial: 72nd annual meeting of the Italian society of physiology: new perspectives in physiological research

**DOI:** 10.3389/fphys.2024.1403715

**Published:** 2024-04-05

**Authors:** Andrea Gerbino, Grazia Tamma, Fiorenzo Conti, Giovanna Valenti

**Affiliations:** ^1^ Department of Biosciences, Biotechnologies and Environment, Università di Bari Aldo Moro, Bari, Italy; ^2^ Section of Neuroscience and Cell Biology, Department of Experimental and Clinical Medicine, Università Politecnica delle Marche, Ancona, Italy

**Keywords:** autopaghy, aging, Ca^2+^ siganling, herbal extracts, intercellular signaling, oxidative stress

The 72nd Annual Meeting of the Italian Society of Physiology, held in Bari in September 2022, provided a vibrant platform for the convergence of leading physiologists, fostering interdisciplinary dialogue, and paving the way for innovative research directions. This Research Topic encompasses a diverse array of topics that emerged from the rich texture of discussions and presentations at the conference. Gathering insights from esteemed researchers across Italy, the Research Topic delves into groundbreaking studies that shed light on various facets of physiological research. Covering a spectrum of themes ranging from cell physiology to neurobiology and cardiovascular health, each article offers a unique perspective, contributing to the collective understanding of complex biological processes.

In exploring novel therapeutic approaches for neurological disorders, the spotlight on autophagy emerges as a promising avenue. Traumatic brain injury (TBI) poses significant challenges in treatment, often leaving limited options due to its complex pathophysiology. However, recent investigations into the role of autophagy machinery, particularly in post-TBI neuronal responses, shed light on potential interventions. Boswellia Sacra gum resin (BSR) emerges as a notable candidate, exhibiting modulatory effects on neuronal autophagy and demonstrating substantial improvements in functional recovery within a mouse model of TBI (Interdonato et al.). This aligns with a broader narrative within the field of neurodegenerative diseases, notably Parkinson’s disease (PD), where a repurposing strategy involving Type 2 Diabetes Mellitus (T2DM) drugs gains support. The interplay between T2DM and PD pathogenesis, marked by disruptions in autophagic processes, underscores the therapeutic potential of antidiabetic medications (Greco et al.). By restoring autophagic function, these drugs offer a glimmer of hope in mitigating neurodegenerative processes and enhancing neuronal resilience. Thus, the convergence of research efforts in different fields highlights the pivotal role of autophagy modulation in addressing multifaceted challenges within the complex scenario of neurological disorders. As the population ages, the incidence of neurological diseases tends to increase. This demographic trend underscores the importance of developing accurate and reliable instruments for monitoring individual health, particularly among the elderly. A recent study developed a composite measure of fitness status based on multiple tests, including the six-minute walking test. Data from eight fitness tests were collected from 176 participants aged 51–80. The newly developed biomarker for biological aging showed strong associations with cardiovascular risk scores and mortality predictions, outperforming previous methods (Manca et al.). This approach has potential for clinical screening and monitoring but requires further validation.

Exploring the therapeutic potential of botanical extracts and dietary supplements unveils a compelling narrative in addressing various health challenges. An anthocyanin-rich fraction extracted from *Callistemon citrinus* flowers emerges as a protective shield against oxidative stress-induced damage in human red blood cells (RBCs). By preserving RBC morphology and anion exchanger 1 (band 3, SLC4A1/AE1) activity, this extract underscores the importance of dietary interventions in fighting oxidative stress-related pathologies (Remigante et al.). Similarly, niacin combined with citicoline demonstrates promise in restoring retinal physiology preventing retinal ganglion cell loss, offering a glimmer of hope in the management of hypertensive glaucoma (Melecchi et al.). Furthermore, bergamot extract emerges as a potent anti-aging agent, exhibiting profound effects on human RBCs exposed to D-Galactose-induced aging (Remigante et al.). Through its multifaceted antioxidant and metabolic regulatory properties, bergamot extract holds potential in mitigating age-related changes, thus presenting a novel avenue in anti-aging therapeutics. Collectively, these findings underscore the burgeoning interest in harnessing the therapeutic power of botanical extracts and dietary supplements in addressing a spectrum of health disorders.

Resolving the intricate web of cellular communication unveils a captivating narrative in understanding disease pathogenesis and therapeutic interventions. In the context of neurodegenerative disorders like Amyotrophic Lateral Sclerosis (ALS), the crosstalk between microglia, astrocytes, and infiltrating immune cells emerges as a pivotal player in shaping the neuroinflammatory milieu (Calafatti et al.). By orchestrating a pro-inflammatory microenvironment, this intricate interplay exacerbates neuronal damage, further fueling disease progression. Meanwhile, tunneling nanotubes (TNTs) offer a novel avenue for long-range intercellular communication within the central nervous system (CNS), yet this area remains largely unexplored (Capobianco et al.). Serving as conduits for the exchange of small signals and large cargo between CNS cells, TNTs redefine our understanding of cellular interactions in controlling CNS functions. As we delve deeper into these intercellular dialogues, new therapeutic strategies aiming to modulate microglial phenotypes and restore CNS homeostasis are on the horizon, holding promise in mitigating neurodegenerative processes and improving patient outcomes.

The deleterious impact of environmental pollutants on human health is a growing concern, with airborne particulate matter less than 10 µM in size (PM 10) emerging as a significant risk factor. Investigations into the cellular mechanisms underlying PM-induced cytotoxicity shed light on its role in triggering apoptotic volume decrease (AVD), a hallmark of early apoptosis, in A549 pulmonary cells (Giordano et al.). Furthermore, chronic exposure to glyphosate, a common herbicide, and its metabolite AMPA, raises questions about their potential cardiovascular effects. Studies elucidating the biological effects of sub-lethal doses of these agrochemicals on H9c2 cardiac myoblasts uncover a complex interplay between these agrochemicals and cytotoxicity (reduction in cell viability, increased ROS production, morphological alterations, and mitochondrial dysfunction), highlighting the need for further research to inform regulatory policies and public health interventions (Arrigo et al.).

Finally, the dynamic orchestration of intracellular signaling pathways lies at the heart of cellular function and disease pathogenesis. Despite decades of research, the spatiotemporal analysis of Ca^2+^ signaling events continues to surprise, revealing previously unexplored facets of cellular function. The experimental exploitation of classic and newly developed genetically encoded fluorescent probes is opening new windows, shedding light on unexpected roles for this ubiquitous ion (Moccia et al.). Recent investigations have elucidated four such roles: 1) the transient receptor potential mucolipin 1 (TRPML1) channel plays a crucial role in modulating water reabsorption in the kidney; 2) dysregulation of endoplasmic reticulum-to-mitochondria Ca^2+^ transfer contributes to astroglial dysfunction in Alzheimer’s Disease; 3) TRP Melastatin 8 (TRPM8) plays a non-canonical role as a Rap1A inhibitor in cancer progression; and 4) non-genetic optical stimulation might serve as a new tool to enable precise manipulation of Ca^2+^ signals in cardiovascular function. In addition, investigations into the effects of Type 2 Diabetes Mellitus (T2DM) on vascular smooth muscle cells (VSMCs) have uncovered significant alterations in intracellular Ca^2+^ handling (Moreno-Salgado et al.). Studies conducted in Zucker Diabetic Fatty rats have shown that T2DM leads to decreased Ca^2+^ release from the sarcoplasmic reticulum (SR) and increased activity of store-operated channels (SOCs) in VSMCs. Furthermore, enhanced cytosolic Ca^2+^ activity during the early stage of ATP-induced Ca^2+^ transient decay, along with alterations in the activity of Ca^2+^ extrusion mechanisms, suggests a potential link between dysregulated Ca^2+^ homeostasis in VSMCs and vascular dysfunction associated with T2DM.

As we navigate the ever-expanding landscape of physiological research, it is essential to acknowledge the collaborative efforts of scientists, clinicians, and educators driving scientific innovation forward. The 72nd Annual Meeting of the Italian Society of Physiology exemplifies the spirit of inquiry and collaboration that fuels progress in biomedical sciences. We extend our heartfelt gratitude to all the authors, reviewers, and contributors who have made this Research Topic possible.

In conclusion, this Research Topic serves as a demonstration to the boundless curiosity and collective endeavor of the scientific community. May the discoveries unveiled within these pages inspire future generations of physiologists and propel us closer to unraveling the mysteries of human biology and health.

